# Mummy Prevents IL-1β-Induced Inflammatory Responses and Cartilage Matrix Degradation via Inhibition of NF-қB Subunits Gene Expression in Pellet Culture System

**DOI:** 10.15171/apb.2018.033

**Published:** 2018-06-19

**Authors:** Fereshteh Morrovati, Nahid Karimian Fathi, Jafar Soleimani Rad, Azadeh Montaseri

**Affiliations:** ^1^Stem Cell Research Center, Tabriz University of Medical Sciences, Tabriz, Iran.; ^2^Biochemistry Department, Faculty of Medicine, Tabriz University of Medical Sciences, Tabriz, Iran.

**Keywords:** Chondrocyte, Inflammatory response, IL-1β, Pellet culture, Cartilage

## Abstract

***Purpose:*** In Persian traditional medicine, application of Mummy material has been advised since hundred years ago for treatment of different diseases as bone fracture, cutaneous wounds and joint inflammation. Regarding to the claim of indigenous people for application of this material in the treatment of joint inflammation, the present study was designed to evaluate whether Mummy can revoke the inflammatory responses in chondrocytes stimulated with interleukin 1-β (IL-1β).

***Methods:*** Isolated chondrocytes at the second passage were plated in 50 ml conical tubes at density of 1x10^6^ for pellet culture or were plated in T75 culture flasks as monolayer. Cells in both groups were treated as control (receiving serum free culture medium), negative control (receiving IL-1β (10ng/ml for 24 hr)) and IL-1β pre-stimulated cells which treated with Mummy at concentrations of 500 and 1000µg/ml for 72hrs. After 72 hrs, to evaluate whether Mummy can revoke the inflammatory response in chondrocytes, cell in different groups were prepared for investigation of gene expression profile of collagen II, Cox-2, MMP-13, C-Rel and P65 using real-time RT-PCR.

***Results:*** Treatment of chondrocytes with IL-1β (10ng/ml) resulted in a significant increase in expression level of Cox-2, MMP-13, C-Rel and P65 in pellet culture system, while treatment of IL-1β-stimulated choncrocytes with Mummy at both concentrations of 500 and 1000µg/ml inhibited the expression level of above mentioned genes. Compared to the pellet culture, Mummy did not affect expression level of genes in monolayer condition.

***Conclusion:*** The obtained data from this investigation revealed that Mummy can be used as a potent factor for inhibiting the inflammatory responses induced by IL-1β in chondrocytes probably through inhibition of NF-қB subunits activation.

## Introduction

Articular cartilage is basically composed of unique cell type named chondrocytes which are trapped in a delicately organized extracellular matrix (ECM).^1,2^ Despite of low division activity, chondrocytes are responsible for maintaining the homeostasis in cartilage tissue through regulation of ECM components synthesis and catabolism.^3^

Articular cartilage ECM is particularly formed by macromolecules such as type II collagen, proteoglycans and associated water which provide tensile strength and flexibility for normal function.^3,4^

Due to some constitutional properties such as lack of vessels, nerve and lymphatics and limited proliferative capacity of chondrocytes, articular cartilage has very poor ability for self- regeneration which leads to gradual impairment of joint tissues.

Osteoarthritis (OA) which is the most common debilitating and chronic disorder of synovial joints, is characterized by progressive loss of articular cartilage and involvement of other joint tissues including subchondral bone and synovium.^5,6^

At molecular level, over production of pro-inflommatory cytokines such as IL-1β and TNF-α by stimulated synoviocytes and chondrocytes has a crucial function in osteoarthritis pathogenesis.^7,8^ Elevated IL-1β levels upregulates the production of degrading enzymes such as matrix metallopreteinases (MMPs) and downregulates the ECM molecules synthesis by chondrocytes which finally results in degradation of articular cartilage.^7,9^ Furthermore, local synthesis and release of IL-1β induces the production of inflammatory pain mediators such as Cox-2 and inducible nitric oxide synthase (iNOS) which leads to clinical symptoms of OA.^10,11^

The biological effects of pro-inflammatory cytokines on chondrocytes such as increased level of Cox-2 and MMPs, are mainly excreted by activation of transcription factor NF-қB.^7,10^ which is the master regulator of several genes involved in inflammation, apoptosis and proliferation.^11,12^

NF-қB is an ubiquitous transcription factor located in the cytoplasm, in its inactive form this molecule is a heterodimer complex consists of two subunits named as P65 and P50 and an inhibitory IқBα subunit.^11^ Different stimulants such as pro-inflammatory cytokines can trigger the phosphorylation and subsequent degradation of IқBα which results in translocation of other subunits to the nucleus, where they can bind to the promoter regions of target genes and induce their transcription.^5,13^

In recent decades, transplantation of *in vitro* expanded chondrocytes at the lesion site has been applied to restore damaged cartilage. The drawback for application of chondrocytes is that during *in vitro* expansion, dedifferentiation of these cells occurs which results in loss of phenotype and also ECM components such as type II collagen, glycosaminoglycans and aggrecan. So, it is becoming increasingly believed that transfer of chondrocyte to the pellet culture systems can extend the redifferentiation capacity of these cells.^14^

Conventional options for treatment of OA are non steroidal anti inflammatory drugs (NSAIDs) and Cox-2 inhibitors which can control the symptoms of disease such as pain and stiffness.^9^ Even so, due to serious side effects of above mentioned agents, it is of great clinical importance to identify novel natural compounds which can be safely applied as an alternative therapy for OA.^15^

In context of traditional Persian medicine, Mummy which is called also Mumnyae by some indigenous people is being used for hundreds of years for healing of joint inflammation, bone fractures and wound healing.^16^ This pitch-like material is dark brown to black in color and formed as a result of oil oxidation in cracks of some caves. Mummy contains different ions including phosphate, calcium, hydrocarbons, polysaccharides and also nitrogen. Recently the potential effects of Mummy for healing of cutaneous wounds, peptic ulcer and bone fractures has been investigated by some researcher in Iran.^16,17^ The unpublished data of our research group also revealed that Mummy can promote the proliferation rate of chondrocytes and also up-regulates the expression of some cartilage-specific genes in *in vitro* condition.

In consideration to the Mummy effects for treatment of joint inflammation which is expressed by local people, in the present study we designed to understand if Mummy can suppress the increased amount of MMP-13 and COX-2 in IL-1β-induced chondrocytes and whether Mummy can affect the expression of transcription factor NF-қB in stimulated cells.

## Materials and Methods

### Cartilage sampling and chondrocyte isolation

Cartilage samples were obtained from patients underwent to the total hip joint replacement surgery due to femoral neck fracture.

Well-preserved cartilage samples were transferred to the culture lab, after washing three times with phosphate buffer saline (PBS)(Cat No: P4417, Sigma-Aldrich, Germany) containing 1% penicillin/ Streptomycin (P/S)(Cat No:ATRA-010, ATOCEL, Hungary), samples were cut into 1x1 mm thickness pieces using sterile scalpel for isolation of chondrocytes, obtained pieces were incubated in pronase 1%(Cat No:10165921001,Roche, Germany) for 60 minutes, further digestion was performed by incubation in collagenase enzyme (Cat No:11088807001,Roche, Germany) at concentration of 0.2% in shaking water bath for 3-4 hours. After enzymatical digestion, undigested particles were removed by filtering through nylon mesh cell strainer (70 µm) and obtained cell suspension was centrifuged at 1500 rpm for 10 minutes. Chondrocytres were counted and seeded in T75 culture flasks containing Dulbecco's Modified Eagle Medium (DMEM)(Lot No:013BS536, biosera, France) supplemented with 10% fetal bovine serum (FBS)(Cat No:10270,Gibco, USA ) and 1% P/S. After reaching about 70% confluency cells were passaged using trypsin/ EDTA enzyme.(Cat No:L0940-100, Biowest, France)

### Preparation of Mummy

Mummy was purchased from local botanical market in Kermanshah, Iran. The effective concentration of this material on chondroytes has been previously determined using MTT assay technique by our Research group,^18^ so in the present study, Mummy was used at concentration of 500 and 1000 µg/ml. Due to the water solubility of this material, Mummy was dissolved in DMEM culture medium and sterilization was done by filtering through syringe filter (0.22 µm).

### Experimental design

Chondrocytes at the second cell passage were used for this study. For pellet culture, cells were put at concentration of 1x10^6^ in 50ml conical tubes with filtered lids. After centrifugation, cell pellets were formed. Furthermore, monolayer cell culture was also performed by seeding 5x10^5^ cells in T75 culture flasks. Chondrocytes in both pellet culture and monolayer condition were cultured in whole culture medium containing 10% FBS for 24hr. To understand the effects of Mummy on inflamed chondrocytes, the cellular events occurred in clinical condition of OA were mimicked by chondrocyte stimulation using IL-1β. For this purpose, chondrocytes in both monolayer and pellet culture were washed with DMEM containing 0.05% FBS before being treated with IL-1β. Serum-starved chondrocytes in both monolayer and pellet culture divided into different groups as below: in the first group (control) cells received only DMEM culture medium (containing 0.05% FBS), in the second group (negative control) cells received DMEM containing 10 ng/ml IL-1β (Cat No: 19401, sigma-Aldrich, USA), cells in the third group received Mummy at concentrations of 500 or 1000µg/ml, and in the fourth group, cells were pre-stimulated for 24 hr using IL-1β (10ng/ml) and then treated with Mummy for additional 72 hrs. After this period, cells in different groups were prepared for further evaluation of gene expression using Real time RT-PCR technique.

### Real time RT-PCR for gene expression

The genetic information for type II collagen, Cox-2, MMP-13 and NF-қB subunits including C-Rel and P-65 in all different groups was analyzed using Real time Reverse transcriptase (RT) PCR. For this purpose, firstly the total RNA content was extracted using YTA mini kit (Cat. No: YT9065, Taiwan) based on the manufacture's instruction.

Briefly, for cell lysis, RB buffer was added and then obtained mixture was transferred to the collection tube containing filter column. After centrifugation, 70% ethanol was added and then two subsequent washings were performed.

To obtain RNA, in the last step RNase-free dd H_2_o was added to samples. About 1000 ng of total obtained RNA was used for CDNA synthesis using cDNA synthesis kit (Cat.No: YT4500, Taiwan). The Real time PCR reactions were performed using PCR Rotor Gene 6000 (Corbett, 010755, Australia) with SYBR green PCR master mix (Takara, RR & 20L, Japan). Different time and annealing temperatures were adjusted for each gene which are listed in Table 1.

**Table 1:** Primer sequences used in Real Time RT-PCR and related annealing temperature.

**Gene**	**Primer pair sequence (5'à3')**	**Annealing temperature**
**hCOL2B**	Forward primer AGGGCCAGGATGTCCGGCA Reverse primer GGGTCCCAGGTTCTCCATCT	‏°‏59C for 30 sec
**COX2**	Forward primer CACCGACTACGGCGGACTAA Reverse primer ACGTCAAGGAGTCGCAGGTC	57°C for 30 sec
**MMP13**	Forward primer CGGCTTAGAGGTGACTGGCA Reverse primer TCAGGAACCCCGCATCTTGG	‏°‏59C for 30 sec
**c-Rel**	Forward primer GAATCAATCCATTCAATGTCCC Reverse primer AAGAGCAGTCGTCAAATTACC	56°C for 30 sec
**P65**	Forward primer AGCACAGATACCACCAAGAC Reverse primer TCAGCCTCATAGAAGCCATCC	56°C for 30 sec
**GAPDH**	Forward primer CCATGTTGCAACCGGGAAGG Reverse primer AGGAGCGCAGGGTTAGTCAC	58°C for 45 sec

### Statistical analysis

Statistical difference between different groups was determined by Two-Way ANOVA method and followed by t-test post test. The results are shown as the mean ± SD of a representative experiment performed three times. Data shown are representative of three independent experiments.

## Results

### Mummy enhances the expression of type II collagen by chondrocytes in both monolayer and pellet culture system

To evaluate whether Mummy material can enhance the mRNA expression level of type II collagen, chondrocytes in both monolayer and pellet culture systems were treated with Mummy at concentration of 500 and 1000 µg/ml. Furthermore to understand if Mummy can revoke the effects of IL-1β on chondrocytes, cells were stimulated with IL-1β (24 hr) and then treated with Mummy at above mentioned concentrations.

**Figure 1:**
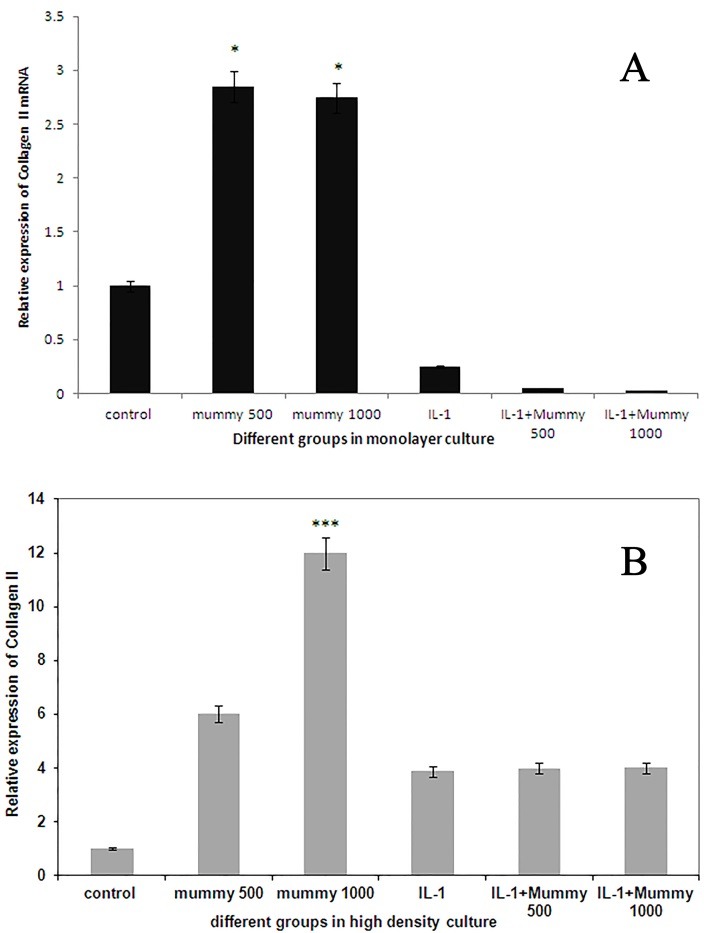
Effect of Mummy material on collagen type II expression level in A) monolayer and B) pellet culture system. as it can be found treatment of chondrocytes with Mummy at 1000mg/ml resulted in a significant increase in expression level of collagen type II gene in both monolayer and pellet culture systems with *P<0.05, ***P<0.0005, respectively.

As it can be found in Figure 1-A, the expression of collagen II in chondrocytes treated with Mummy in both concentration of 500 and 1000 µg/ml elevated significantly compared to the control cells (p<0.05), but there isn't any significant difference between expression level of collagen type II in cells treated with IL-1β compared to control cells (p<0.4).

Figure 1-B shows the expression level of collagen type II in chondrocytes cultivated as pellet culture. As it can be found, Mummy at concentration of 1000 µg/ml enhanced the level of type II collagen compared to the control cells (p<0.0005) but there isn't any significant difference in mRNA expressin level of collagen II between IL-1β treated groups and control cells (p<0.5).

### Mummy can down regulate the elevated levels of COX-2 and MMP-13 in IL-1β-stimulated chondrocytes

**Figure 2:**
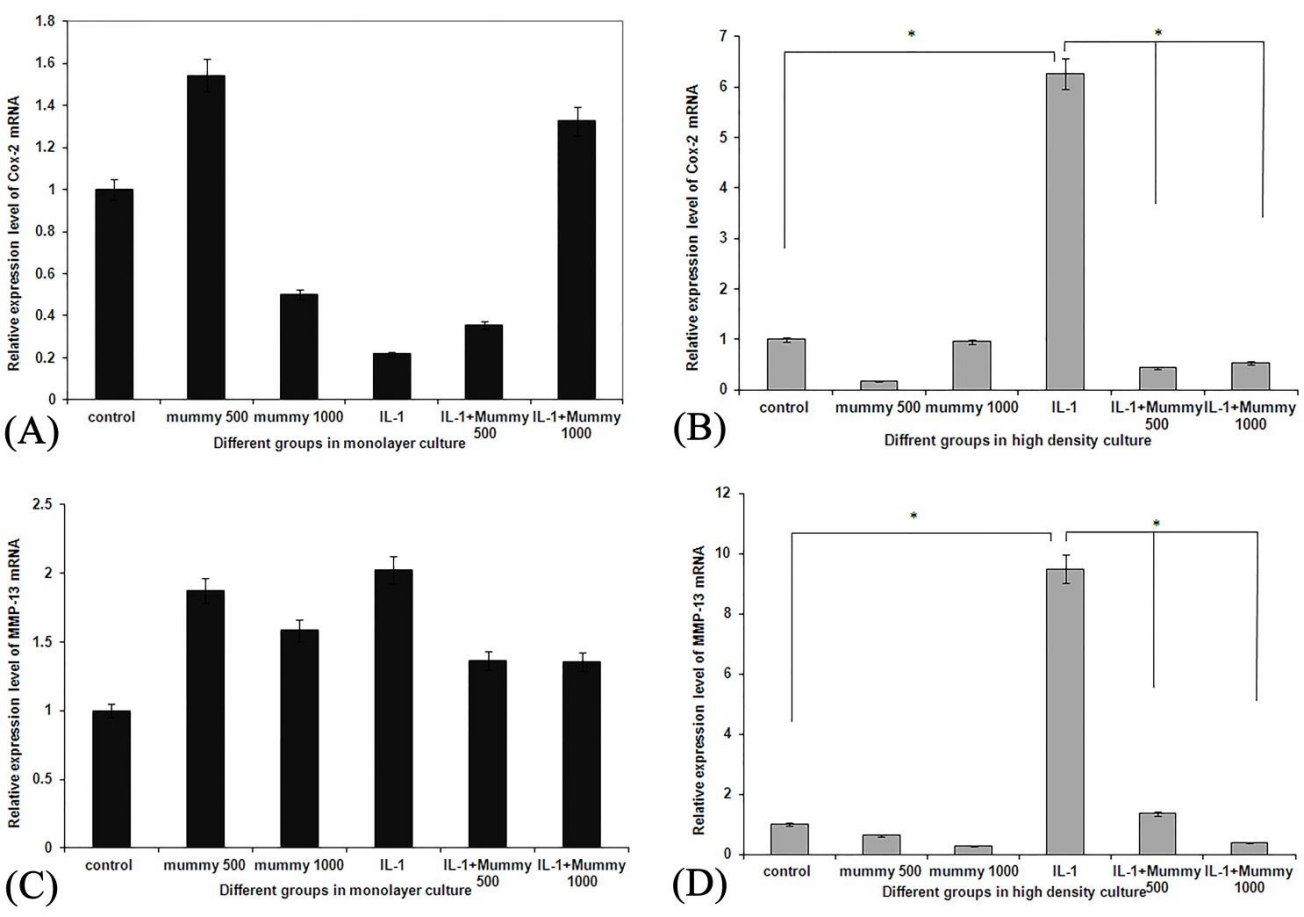
mRNA expression level of Cox-2 gene in A) monolayer and B) pellet culture systems, expression level of MMP-13 mRNA in C) monolayer and D) pellet culture conditions. Treatment of primary chondrocytes in monolayer culture did not affect the expression level of Cox-2 and MMP-13, but as it can be understood from figures 2-B and D, the expression level of both genes in pellet culture decreased significantly after treatment of IL-1β-stimulated chondrocytes with Mummy in both concentrations. *P<0.05

To evaluate whether Mummy can modulate the increased level of COX-2 and MMP-13 in IL-1β-stimulated chondrocytes, at first cells were pre-stimulated with IL-1β (24 hr) and then treated with Mummy (500 and 1000 µg/ml) for additional 72 hrs. As Figures 2-A and C show there isn’t any significant difference in COX-2 and MMP-13 expression level between Mummy treated chondrocytes compared to the control cells in monolayer condition (p<0.6), also as it can be revealed stimulation of cells with IL-1β didn’t affect significantly the COX-2 and MMP-13 expression compared to the control cells or cells treated with Mummy in monolayer culture system (p<0.9).

IL-1β pre-stimulation of chondrocytes in pellet culture system resulted in a significant increase of COX-2 expression level compared to the control or Mummy treated cells (p<0.05) (Figure 2-B). Furthermore as Figure 2-B reveals Mummy treatment of IL-1β-stimulated chondrocytes revoke the elevated level of COX-2 mRNA expression in pellet culture system (p<0.05).

Figure 2-D demonstrates the expression level of MMP-13 in pellet culture cultivated chondrocytes. As it can be understood, Mummy treatment of IL-1β-stimulated cells resulted in a significant down-regulation of MMP-13 compared to the chondrocytes which received only IL-1β (p<0.4) (Figure 2-D).

### Mummy can down regulate the elevated levels of c-Rel and p65 subunits in IL-1β-stimulated chondrocytes cultivated in pellet culture condition

To evaluate whether treatment of chondrocyte with Mummy can change the expression of NF-қB subunits including c-Rel and p65, the mRNA expression level of these genes was assessed in both monolayer and pellet culture conditions.

**Figure 3:**
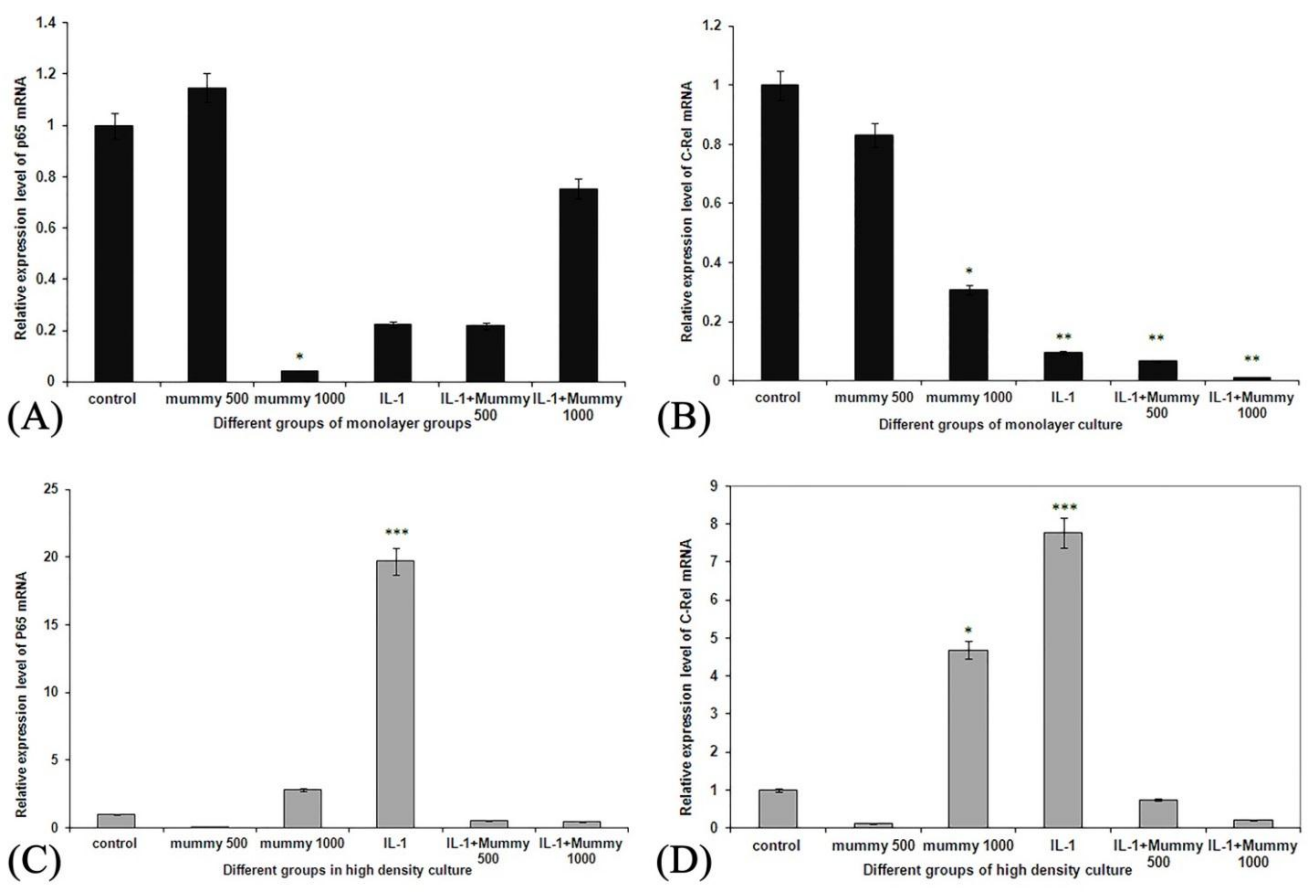
Expression level of P-65 mRNA in A) monolayer and C) pellet culture conditions, and mRNA expression level of C-Rel gene in B) monolayer and D) pellet cultures. As it can be revealed treatment of chondrocytes with IL-1β resulted in a significant increase in expression level of both c-Rel and P65 subunits in pellet culture of cells and treatment of IL-1β-stimulated cells with Mummy at both concentrations decreased the expression level of both mentioned genes significantly. *p<0.05, **p<0.005, ***p<0.00053

Figure 3-A shows the expression level of p65 genes in monolayer cultivated cells, as it can be understood stimulation of chondrocytes with IL-1β and also treatment of IL-1β-stimulated chondrocytes with Mummy didn’t change the expression level of this gene significantly (p<0.1). Additionally the expression level of c-Rel as another subunit of NF-қB decreased significantly after treatment of monolayer-cultivated chondrocytes with IL-1β (p<0.005) compared to the control cells and treatment of IL-1β-stimulated chondrocytes with Mummy at both concentrations resulted in a significant change in the expression level of c-Rel compared to control cells (p<0.001) but not in comparison with IL-1β-stimulated cells (p<0.9) (Figure 3-C).

Figures 3-C and D shows the expression level of both c-Rel and p65 genes in pellet culture of chondrocytes.

As it can be understood stimulation of chondrocytes with IL-1β resulted in a significant increase of both NF-KB subunits compared to the control (p<0.005). Furthermore treatment of IL-1β-stimulated chondrocytes with Mummy at both 500 and 1000 µg/ml inhibited significantly the expression level of both c-Rel and p65 genes (p<0.0005).

## Discussion

This study found the following results: 1) Mummy treatment of chondrocytes in monolayer and pellet cultures increased the expression of type II collagen genes, 2) IL-1β stimulation resulted in upregulation of p65 and c-Rel subunits in chondrocytes cultivated in pellet culture which is reversed significantly after treatment with Mummy, 3) Mummy treatment of chondrocytes in pellet culture condition revokes IL-1β-induced upregulation of cox-2 and MMP-13.

One of the most common debilitating diseases affecting large number of elders is osteoarthritis (OA). This active and dynamic disease affects all joint tissues and results in structural and functional impairment of articular cartilage and remodeling of underlying bone. Due to the avascular characteristic of articular cartilage and low proliferative potential of chondrocytes, this tissue has a limited ability for self-regenaration.^11^

Current pharmacologic therapies for treatment of OA usually are alleviative and cannot prevent the progressive degradation of articular cartilage,^19^ furthermore long term consumption of these drugs normally is associated with severe side effects.^20^ As a consequence, identifying novel complementary medicines with more safety and effectiveness is of great importance.^9^

In Persian traditional medicine Mummy material has been advised since hundreds of years for treatment of different diseases such as joint inflammation, wound healing and bone fractures. Recently, several investigations revealed some pharmacological effects of Mummy such as anti-inflammatory, promoting collagen production at wound site, bone fracture healing and protection of gastric mucosa against ethanol-induced ulcer.^16,17^ The molecular mechanisms underlying the therapeutic effects of Mummy in joint inflammation remains unclear, hence this study was designed to evaluate if Mummy can revoke degenerative effects of IL-1β on chondrocytes.

The widely accepted process involved in initiation and advancement of OA includes degradation of articular cartilage, inflammation of synovial membrane and changes in subchondral bone.^8^

Pro-inflammatory cytokines such as IL-1β produced by synoviocytes and chondrocytes are largely participating in OA pathogenesis.^9^ Elevated amount of IL-1β results in upregulation of Matrix metaloproteinnases (MMPs), cox-2 and Nitric oxide (NO) which are regulated by the ubiquitous transcription factor, nuclear factor-КB (NF-КB).^5,7^

In unstimulated state, this factor is located in cytoplasm and combined with an inhibitory subunit named as IKBα. Due to IL-1β stimulation, activated NF-қB subunits translocate to the nucleus and bind to the promoter regions of different inflammation-related genes including MMP-1, MMp-9, MMP-13, Cox-2, NO and different types of ADAMTS which finally result in further degradation of cartilage ECM components such as type II collagen and proteoglycans.^10,21^

It has been well accepted that in *in vitro* conditions, stimulation of chondrocytes with IL-1β at concentration of 10 ng/ml can mimic pathological events of OA.^22^

In the present study for understanding the probable effects of Mummy on inflammatory pathway occurred in OA, IL-1β-stimulated chondrocytes were used.

As we found, the gene expression level of two NF-қB subunits including c-Rel and p65 in the presence of IL-1β increased significantly in chondrocytes cultivated in pellet culture system. For evaluating the effects of Mummy, IL-1β-stimulated chondrocytes were treated with Mummy at both 500 and 1000 µg/ml doses. This investigation reveals that the expression level of both c-Rel and p65 subunits decreased significantly after Mummy treatment in IL-1β-pre stimulated chondrocytes.

Also, we found that IL-1 stimulation results in upregulation of MMP-13 and Cox-2 genes in pellet culture cultivated chondrocytes and then treatment of IL-1β-stimulated cells with Mummy (500, 1000 µg/ml) inhibit their upregulation. As it has been previously mentioned, due to activation of NF-қB, synthesis of matrix degrading enzymes such as MMPs, Cox-2 and NO levels increase.

In normal conditions, there is a delicately regulated balance in ECM component remodeling through MMP activity and synthesis of new matrix.^7^ However, during OA, this equilibrium gets lost and expression of different MMPs such as MMP-13 enhances which plays a crucial role in collagen type II degradation and cartilage destruction.^3^

Our results reveal that Mummy (500 and 1000 µg/ml) inhibited the IL-1β-induced MMP-13 upregulation, so further investigation are necessary to understand the exact underlying mechanism of this material.

Another inflammation-realated gene which its expression is upregulated due to activation of IL-1β, is cox-2.^7^ This inflammatory pain mediator exerts excessive catabolic effects through prostaglandinE2 (PGE2) production. In this study we found that IL-1β-induced upregulation of Cox-2 can be revoked by Mummy at both concentrations. It could be suggested that down-regulation of MMP-13 and Cox-2 in Mummy-treated chondrocytes occurs via inhibition of two NF-қB subunits including c-Rel and p65, but for understanding the precise underlying mechanisms, further evaluations are needed.

In this study, the probable effects of Mummy on IL-1β-stimulated chondrocytes were evaluated in both monolayer and pellet culture systems. Our results showed that Mummy exerts its anti-inflammatory effects on chondrocytes cultivated in pellet culture condition, rather than monolayer.

It is well established that monolayer expansion of chondrocytes cannot support their phenotype due to the dedifferentiation process, whereas 3D culture can maintain the chondrogenic potential maybe in part as a result of low oxygen tension which occurred normally in cartilage tissue due to its avascularity.^2^

Taking together, in this investigation we demonstrated that treatment of IL-1β-stimulated chondrocytes can inhibit the expression of inflammation-related genes including MMP-13 and cox-2 probably through down-regulation of NF-KB subunits including c-Rel and p65. Further evaluations are necessary for better understanding the precise underlying mechanisms.

## Conclusion

It can be concluded that Mummy can inhibit the inflammatory responses in chondrocytes induced by IL-1β, so it can be introduced as a useful compound with the potential shedding light on advancing the success in cartilage tissue engineering.

## Acknowledgments

This article is resulted from the research proposal leading to thesis of Fereshteh Morovvati, M.Sc student of Anatomical Sciences and approved by Stem Cell Research center, Tabriz University of Medical sciences, Tabriz, Iran. The authors gratefully acknowledge the research deputy of Tabriz University of Medical Sciences for financial support.

## Ethical Issues

Written informed consents were taken from all patients and the study protocol was approved by the institutional review board and medical ethics committee of Tabriz University of Medical sciences (ethical number: IR.TBZMED.REC.1395.398).

## Conflict of Interest

The authors declare no conflict of interests.
